# Oxygenation and Alkalinity Drive the Lacustrine Nitrogen Isotope Record Throughout the Past 3.2 Billion Years

**DOI:** 10.1111/gbi.70033

**Published:** 2025-09-18

**Authors:** Diana Velazquez, Nathan D. Sheldon, Michael T. Hren, Jenan J. Kharbush

**Affiliations:** ^1^ Department of Earth and Environmental Sciences University of Michigan Ann Arbor Michigan USA; ^2^ Department of Earth Sciences University of Connecticut Storrs Connecticut USA

**Keywords:** alkalinity, lake biogeochemistry, nitrogen cycle, nitrogen isotopes, oxygenation

## Abstract

The widespread, stepwise oxygenation of Earth's atmosphere in the Precambrian led to a transformation of the global carbon (C) and nitrogen (N) cycles. While the temporal evolution of these nutrient cycles has been studied extensively in marine environments, lacustrine environments are understudied. This study first examines how water column oxygen conditions impact sedimentary carbon (δ^13^C_org_) and nitrogen (δ^15^N) isotope signals in modern lakes. Subsequently, we use these patterns to interpret past changes in the geological record of lacustrine δ^15^N during atmospheric oxygenation. The compiled modern lake sediment dataset reveals average (± standard deviation) δ^15^N values of +2.9‰ ± 3.2‰ and δ^13^C_org_ values of −25.99‰ ± 3.77‰, as well as thresholds in δ^13^C_org_ for oxic versus anoxic conditions, and in δ^15^N for circumneutral versus alkaline pH conditions. In contrast to the stepwise oxygenation of the atmosphere, the lacustrine δ^15^N record does not directly reflect major oxygenation events, but instead increases gradually in response to the evolution of new aerobic N metabolic pathways, with a notable shift in the Phanerozoic. While we found that intrasite variability at a single modern anoxic lake is expected to remain within ~5‰ for δ^15^N, alkaline lakes in both the ancient and modern deviate from this range. We observe δ^15^N > +10‰ for approximately half of total ancient alkaline lake sediments and some modern lake sediments. This is consistent with previous applications of enriched δ^15^N as a basicity proxy. The lacustrine δ^15^N record aligns well with the evolution of microbial metabolic pathways in addition to providing information pertaining to environmental conditions of the depositional setting.

## 1 Introduction

Nitrogen (N) is an essential nutrient for all living organisms, required alongside carbon (C) for the formation of biological macromolecules. Therefore, tracing N cycling over geological time is crucial for understanding how life evolved in relation to Earth's major environmental changes. The rise of oxygen in Earth's atmosphere subsequently altered aquatic ecosystems and global N cycling by favoring either aerobic or anaerobic metabolic processes and promoting the development of novel N metabolisms (Falkowski and Godfrey 2008; Canfield et al. 2010; Stüeken et al. 2016).

Unlike most of the ocean, where greater depth, volume, and buffering capacity mean that atmospheric changes or allochthonous inputs may take longer to manifest and be recorded in the geological record, lakes are shallower, smaller, and sometimes transient water bodies susceptible to more rapid changes under the same atmospheric conditions. Therefore, lakes could have been one of the first habitats to experience the biogeochemical effects of major atmospheric changes such as increases in atmospheric oxygen levels, and could thus record evidence of the initial shifts in nutrient cycling and microbial communities that followed. Reinforcing the importance of terrestrial environments in our understanding of Earth's oxygenation, there is a growing body of evidence suggesting the presence of terrestrial Archean “oxygen oases”, or local oxidative settings (Lalonde and Konhauser 2015; Sumner et al. 2015), well before the Great Oxidation Event (GOE) ca. 2.45 Ga that marked the rise of oxygen in Earth's atmosphere.

Nitrogen isotopes (δ^15^N) in organic matter within aquatic sediments are a commonly used tool for studying past changes in N cycling because they record shifts in N sources and pathways of transformation (Robinson 2001; Brandes and Devol 2002). Due to the redox sensitivity of N, δ^15^N can also be used as a proxy for environmental oxygenation and redox conditions (Thomazo et al. 2011; Ader et al. 2014, 2016; Stüeken et al. 2016; Cheng et al. 2019; Sigman and Fripiat 2019; Cao et al. 2022). Sedimentary δ^15^N signatures are influenced by N transformations and their associated isotope effects during microbial uptake in the water column and export to sediments. While extensive work has focused on the marine δ^15^N record to understand the evolution of the N cycle alongside critical shifts in Earth's oxygenation (Beaumont and Robert 1999; Falkowski and Godfrey 2008; Busigny et al. 2013; Stüeken et al. 2016; Zerkle and Mikhail 2017), few studies have investigated how oxygenation impacted N cycling in terrestrial aquatic environments such as lakes (Stüeken et al. 2015; Cao et al. 2022; Xia et al. 2022). Lake sediment δ^15^N values have been used to identify alkaline settings (Stüeken et al. 2015; Deng et al. 2018; Xia et al. 2022) and to infer the presence of major N metabolic pathways (Chen et al. 2021; Mercuzot et al. 2021).

The marine δ^15^N sedimentary record appears to be related to the non‐linear progression of Earth's atmospheric and oceanic oxygenation (Stüeken et al. 2016); however, the absence of a step function in the δ^15^N isotope record leaves in question the effect of oxygenation and biological innovation on δ^15^N patterns. In particular, there is no change in the δ^15^N isotope record across the GOE, the greatest shift in redox conditions in Earth history. Instead, a period of anomalously positive δ^15^N values in the marine sedimentary record, termed the Nitrogen Isotope Event (NIE), occurs prior to the GOE around 2.6–2.8 billion years ago. These positive values are hypothesized to originate from partial ammonia oxidation, which would require the presence of oxygen in the water column and therefore oxygenic photosynthesis (Pellerin et al. 2024), possibly sustained by a large upwelled ammonium supply originating from increased hydrothermal activity (Martin et al. 2025).

The lacustrine δ^15^N record offers another opportunity to examine the co‐evolution of N cycling and paleo‐environmental conditions. Although several studies have explored δ^15^N patterns in anoxic lakes (e.g., Busigny et al. 2024), none have conducted cross‐system comparisons to develop a comprehensive understanding of how anoxic water columns influence sedimentary δ^15^N records. Investigating the sedimentary geochemistry of modern anoxic lakes could help fill in this knowledge gap and aid in interpreting ancient lake sedimentary records. In this study, we used both published literature and newly collected samples to compile a dataset comprising geochemical, environmental, and spatial data from modern anoxic and oxic lakes. We use those data to determine how water column redox conditions affect organic matter preservation and, consequently, δ^15^N signals preserved in the rock record. The new δ^15^N data come from Middle Island Sinkhole (MIS), a karst feature in Lake Huron that has low‐oxygen concentrations, benthic microbial mats, and elevated sulfate concentrations resembling mid to late Proterozoic oceans (Rico et al. 2019; Gomes et al. 2022). Here, we use MIS as a model for understanding the extent of geochemical signal variability found within a single anoxic environment. Finally, we reconstruct the lacustrine sedimentary δ^15^N record from published literature, compare it to the marine sedimentary δ^15^N record, and infer global changes in Earth's N cycle and atmospheric conditions, primarily oxygenation.

## 2 Methods

### 2.1 Modern Lake Data Compilation

**FIGURE 1:**
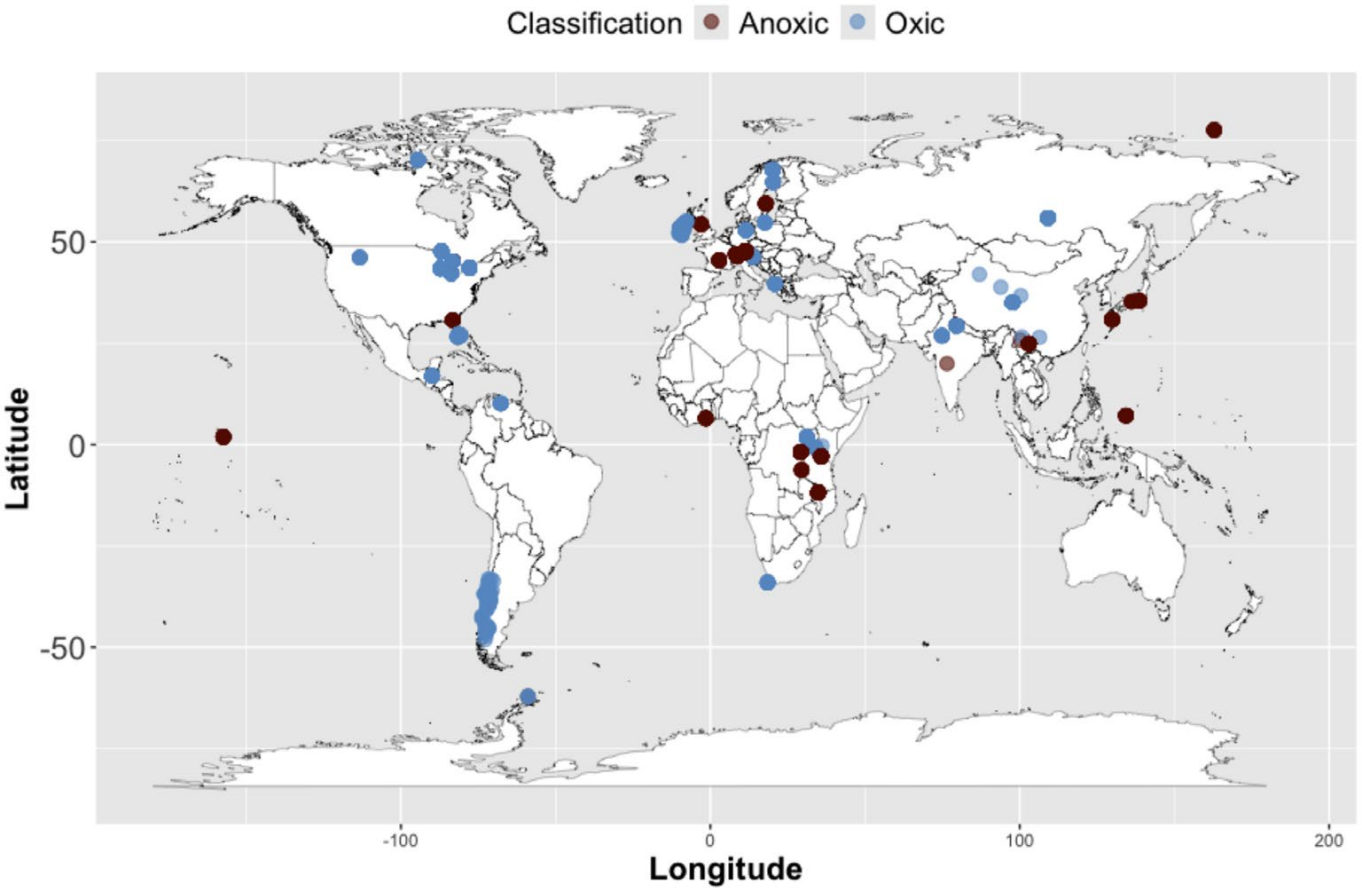
Spatial distribution of modern oxic (*n* = 152; blue) and anoxic (*n* = 27; red) lakes with sedimentary δ^15^N and δ^13^C_org_ data. Alkaline lakes are also included in this map but not specified according to alkalinity.

Modern lake sediment geochemical data were obtained either directly from the publication, through communication with the corresponding author, or extracted from publication figures using WebPlotDigitizer version 4.6 (https://automeris.io/WebPlotDigitizer) when the other options were unavailable. The compiled dataset consists of 179 global lake sites (Figure 1) and includes 27 anoxic lakes. Anoxic lakes are defined as either meromictic, characterized by the absence of oxygen in the monimolimnion due to permanent stratification, or experiencing anthropogenically induced intensification of hypolimnetic anoxia, where the bottom layer of water experiences a lack of dissolved oxygen. Key phrases such as “dissolved oxygen (D.O.) < 2 mg/L”, “meromictic episodes”, and “anoxic bottom‐water conditions” were noted to determine lake classification as anoxic. Trophic status was determined for 84% of the lakes, classifying them as eutrophic, mesotrophic, oligotrophic, or a mixture, as specified in the literature.

While all lakes had complete bulk nitrogen isotope (δ^15^N) and organic carbon isotope data (δ^13^C_org_), a small subset of lakes had complete geochemical and morphological data including percent total nitrogen (%TN), percent total organic carbon (%TOC), atomic carbon to nitrogen (C/N) ratios, total phosphorus (μg P/L), lake type, altitude (m a.s.l.), and surface area (km^2^). Additional publications and/or databases were used when possible if data on parameters of interest were absent from the main study, but all parameters were not available for all published datasets used. Summary data for lake δ^15^N and δ^13^C_org_ values are given in Table 1 and the full dataset is compiled in Data S3 *SI_Modern_Lake_C_N.xlsx*.

**TABLE 1:** Summary of δ^15^N and δ^13^C_org_ values (minimum, maximum, and average ± standard deviation) for ancient and modern lakes, including the total number of data points with both geochemical measurements available.

	δ^13^C_org_ (‰)	δ^15^N (‰)
Ancient lakes (*n* = 1235)		
Min.	−57.06	−2.2
Max.	−15.51	+44.0
Mean ± S.D.	−27.03 ± 4.81	+6.3 ± 5.7
Ancient alkaline lakes (*n* = 264)		
Min.	−57.06	+0.8
Max.	−18.89	+44.0
Mean ± S.D.	−30.23 ± 5.32	+11.4 ± 9.0
Modern lakes (*n* = 1718)		
Min.	−38.72	−21.9
Max.	−10.53	+17.0
Mean ± S.D.	−25.99 ± 3.77	+2.9 ± 3.2
Anoxic lakes (*n* = 796)		
Min.	−38.72	−5.6
Max.	−10.53	+14.5
Mean ± S.D.	−26.47 ± 4.46	+2.5 ± 2.9
Oxic lakes (*n* = 922)		
Min.	−33.36	−21.9
Max.	−16.90	+17.0
Mean ± S.D.	−25.57 ± 3.01	+3.3 ± 3.4
Modern alkaline lakes (*n* = 109)		
Min.	−29.33	+0.2
Max.	−17.31	+17.0
Mean ± S.D.	−23.43 ± 3.18	+6.8 ± 3.79

*Note:* Ancient and modern lakes include all data points corresponding to all lakes regardless of metamorphic grade, alkalinity, or hypolimnetic oxygen conditions. Subsets of the larger ancient and modern datasets are further categorized based on alkalinity (alkaline) or hypolimnetic oxygen conditions (anoxic or oxic). %TN and %TOC for each can be found in Table S1.

### 2.2 Ancient Lacustrine Data Compilation

We identified a total of 39 ancient lacustrine units with published geochemical data for both sediment δ^15^N and δ^13^C_org_ spanning the Paleoarchean (3220 Ma) to the Eocene (45 Ma). Geologic units with debated depositional environments were retained to represent transitional lacustrine settings with possible marine or fluvial influence. The depositional environment, metamorphic grades, and lithologies of lacustrine units were compiled from original publications. While metamorphism can influence δ^15^N values (Thomazo and Papineau 2013), most units exhibit low‐grade metamorphism, ranging from unmetamorphosed to greenschist facies. Only one unit shows a higher metamorphic grade, ranging from lower greenschist to amphibolite facies. Though several studies point to the isotopic differences between nitrogen bound to kerogen (δ^15^N_kerogen_) and nitrogen substituted in potassic minerals (δ^15^N_bulk_), here we have combined both nitrogen fractions given the limited availability of lacustrine δ^15^N data in the literature. Since 93% of the ancient lake data points contain δ^15^N_bulk_, our cross comparisons are consistent. Specifications of kerogen versus bulk δ^15^N values, metamorphic grades, lithologies, and depositional environments corresponding to ancient lacustrine sediments can be found in Data S1 *SI_Ancient‐Lake‐Geochem‐Data3.xlsx* alongside additional geochemical data including %TOC, %TN, and C/N where available. Alkaline lake units were determined based on pH values above 9, as specified by the publication.

### 2.3 Sediment C and N Isotopes From Middle Island Sinkhole, Lake Huron

**FIGURE 2:**
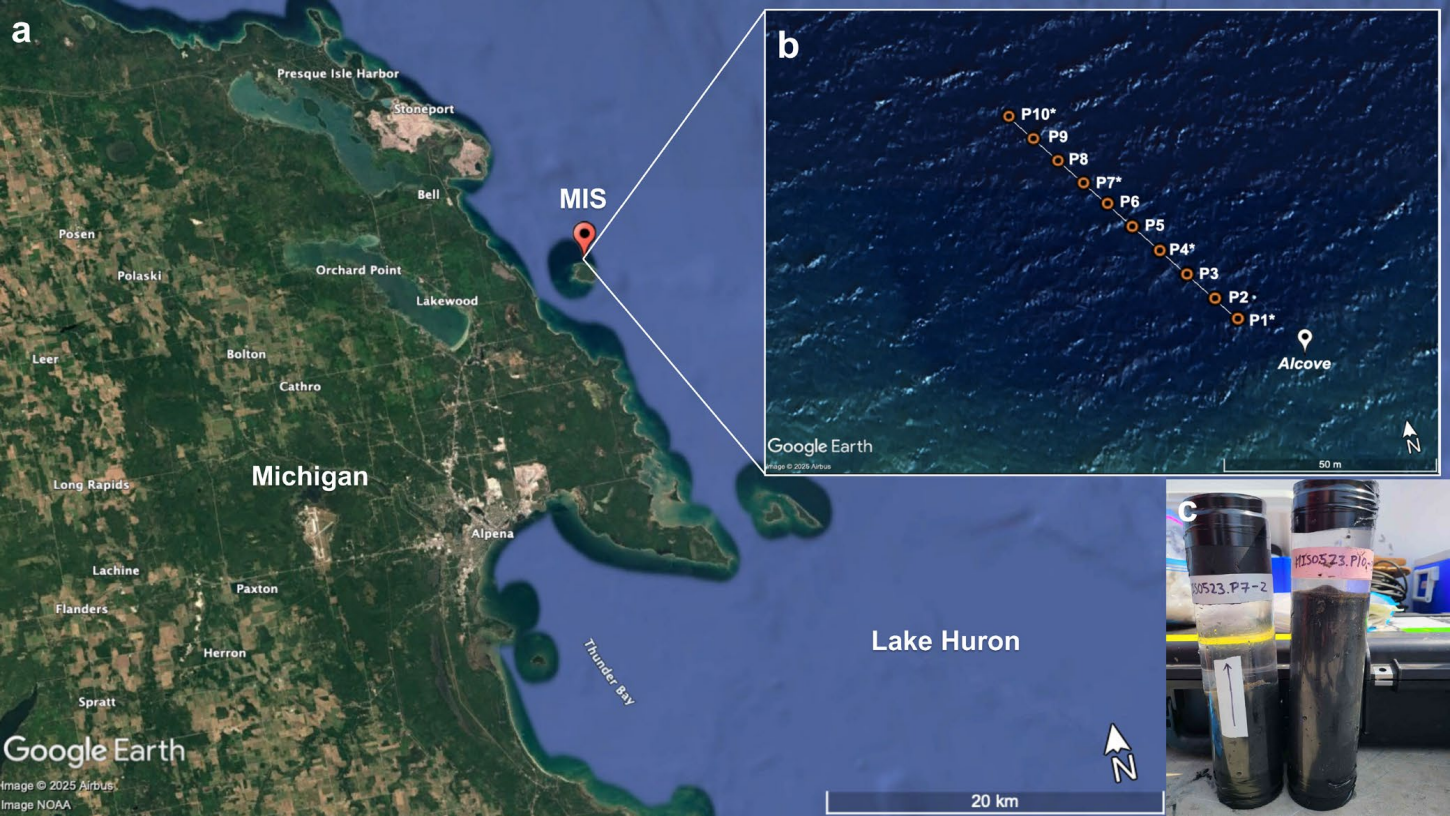
(a) Middle Island Sinkhole (MIS) is located in Lake Huron on the outskirts of Middle Island near Alpena, MI. (b) Sediments were obtained by National Oceanic and Atmospheric Administration (NOAA) Thunder Bay National Marine Sanctuary (TBNMS) divers at four different posts (asterisked) along the sinkhole with varying distances to the alcove, the source of anoxic groundwater. (c) MIS sediments are characterized by a dark, black color reflective of high organic carbon content.

δ^15^N measurements from Middle Island Sinkhole (MIS) included sediment samples collected in 2015, 2016, 2017, and 2021. Samples were collected from the benthic microbial mat/sediment surface up to a depth of 15.5 cm from four sites spanning ~60 m along an established sampling fence line in the sinkhole (Figure 2b). Sampling locations are named according to their distance from the source of anoxic groundwater (known as the alcove), with P1 and P10 as the closest and farthest away, respectively. Upon collection, cores were frozen upright, sectioned (three 1 cm sections at the core top, then 3 cm intervals downcore), freeze‐dried, and homogenized. Decarbonation of samples was performed using a 2% (*w*/*w*) hydrochloric acid solution following the protocol described by Harris et al. (2001). Approximately 10 mL of acid was added to 1 g of homogenized sediment and left to react at 50°C. Acid washes were repeated three times until the reaction stopped to completion (~6 h) to ensure complete removal of carbonates. After each acid wash, samples were rinsed with deionized water and decanted before the next acid treatment. Decarbonated samples were dried at 50°C and weighed (~5–8 mg) into tin capsules. Carbonate contents for MIS sediments ranged from 47.3% to 78.2% (See Data S2 *SI_MIS‐CN‐Isotopes.xlsx*). Decarbonated samples were measured for δ^13^C_org_ and δ^15^N_org_ using a MAT 253 Gas Isotope Ratio Mass Spectrometer coupled to a Costech Elemental Analyzer. Decarbonation of sediments with strong acids has been reported to result in some loss of nitrogen and alteration of δ^15^N (Kennedy et al. 2005; Schlacher and Connolly 2014). To determine potential effects of decarbonation on δ^15^N measurements, 24 of the 167 MIS sediments were also run as untreated sediment for δ^15^N_bulk_, using a Thermo Flash Elemental Analyzer–Delta V isotope ratio mass spectrometer (EA‐IRMS) and standard reference materials USGS40 and USGS41 (See Figure S1). A previous inter‐laboratory comparison of δ^13^C_org_ measurements yielded a slope of 0.96, an *R* ^2^ value of 0.97, and a Pearson correlation coefficient of 0.98, indicating strong agreement between the two instruments (See Data S2 *SI_MIS‐CN‐Isotopes.xlsx*). Acidified and untreated samples were on average 0.8‰ different. Acidified samples were up to −1.7‰ depleted and up to +0.4‰ enriched relative to untreated samples, which is within the range previously reported for ecological samples by Schlacher and Connolly (2014). While acidification therefore affects the absolute δ^15^N values, we primarily use this data to understand the range of intrasite variability.

The Mann Whitney *U*‐test was used to determine statistically significant differences between sampling years and sites for MIS sediment δ^15^N_org_ values. All statistical analyses and plots were created with R Statistical Software (v4.1.2; R Core Team 2021).

## 3 Results

### 3.1 Modern Lake Sedimentary C and N Trends

**FIGURE 3:**
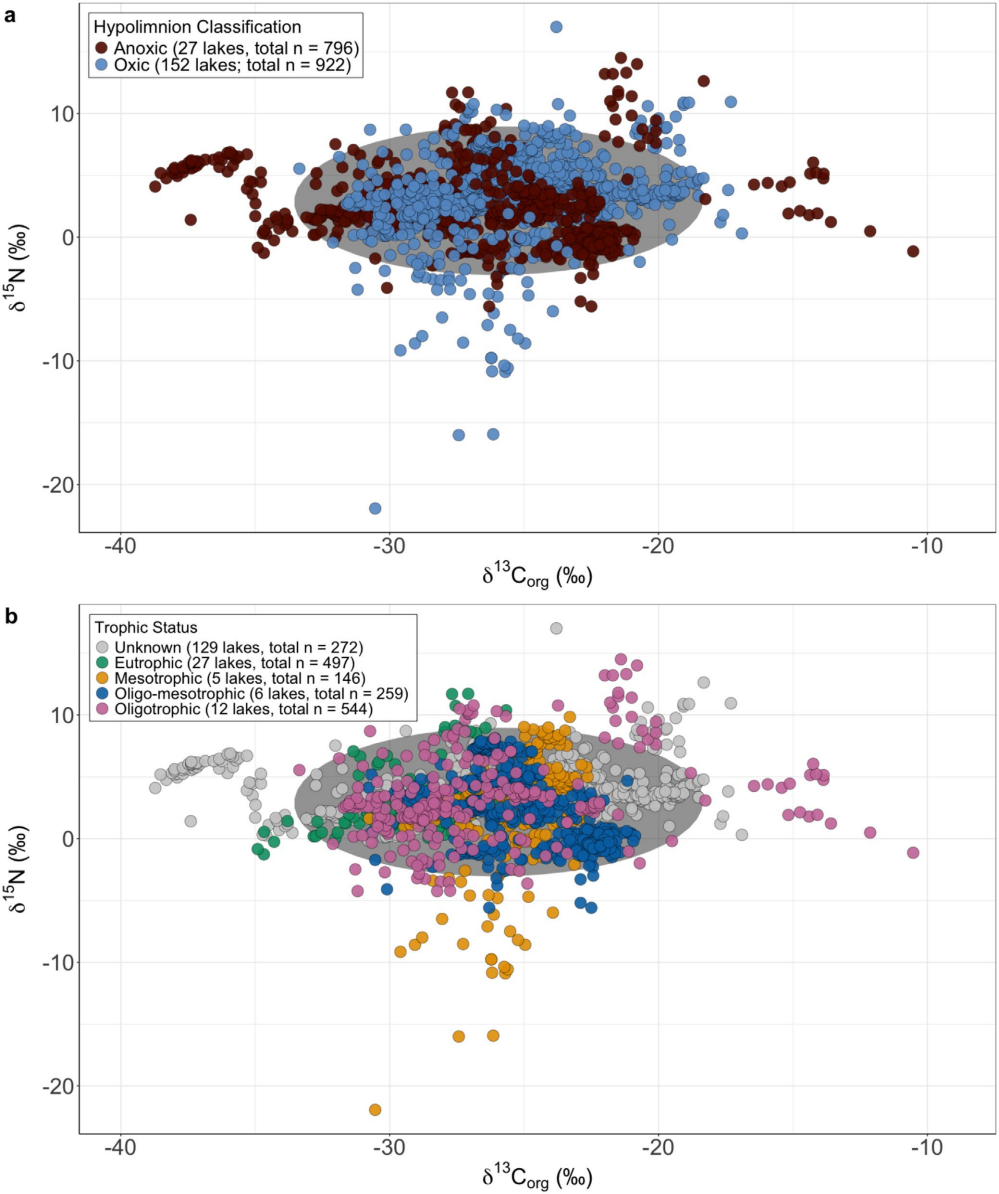
δ^15^N versus δ^13^C_org_ for modern lake sediments (sites *n* = 179, total samples *n* = 1718) (a) separated by bottom water oxygenation and by (b) trophic status, the level of biological productivity of a lake, as specified by the published papers. Lakes were classified as either eutrophic, mesotrophic, oligotrophic, or a mixture. Grey data points represent sediments from lakes whose trophic status was not available in the literature. δ^15^N values include both bulk (δ^15^N_bulk_) and organic fractions (δ^15^N_org_). Deviations outside the shaded grey data array (based on a 95% confidence interval using a multivariate T‐distribution) can be observed for both anoxic (sites *n* = 27, total samples *n* = 796) and oxic (sites *n* = 152, total samples *n* = 922) lake conditions. When considering trophic status, there is no trend between δ^15^N and δ^13^C_org_.

Modern lake data (179 lakes, total *n* = 1718) fall within a range from −21.9‰ to +17.0‰ for δ^15^N and −38.72‰ to −10.53‰ for δ^13^C_org_ (Figure 3a). For the entire modern lake dataset, the average and standard deviation for δ^15^N are +2.9‰ ± 3.2‰ and −25.99‰ ± 3.77‰ for δ^13^C_org_. A Mann Whitney *U*‐test revealed statistically significant differences in the distribution of values between anoxic and oxic lake sediments (*p* < 0.01). On average, anoxic lake sediments exhibit more depleted isotopic compositions, with δ^15^N values of +2.5‰ ± 2.9‰ compared to +3.3‰ ± 3.4‰ in oxic sediments, and values of −26.47% ± 4.46% versus −25.57% ± 3.01%, respectively (Table 1). Additionally, anoxic lake sediments have higher %TOC and %TN than oxic lake sediments (Table S1). The Spearman rank correlation coefficient (*r*_*s*_) was used to assess the strength of relationships between C and N sedimentary geochemical data. In both anoxic and oxic lakes, %TOC and %TN in sediments are strongly correlated (*r*_*s*_ = 0.93, *p* < 0.0001) (Table S2), supporting stoichiometrically non‐selective degradation of organic matter. However, δ^13^C_org_ and δ^15^N show only a very weak correlation (*r*_*s*_ = 0.10, *p* < 0.05), suggesting a potential decoupling between C and N biogeochemical cycling. Similarly, sediment %TOC and δ^13^C_org_ are only weakly correlated (*r*_*s*_ = 0.11, *p* < 0.05), and %TN exhibits a weak negative correlation with δ^15^N (*r*_*s*_ = −0.33, *p* < 0.05). These weak relationships further suggest different controls on C and N cycling in addition to different signals preserved in bulk concentrations compared to isotopic signatures. While redox conditions seem to drive some δ^15^N and δ^13^C_org_ patterns, the trophic status of a lake does not seem to drive isotopic trends (Figure 3b).

### 3.2 Middle Island Sinkhole, Lake Huron Intrasite Variability

**FIGURE 4:**
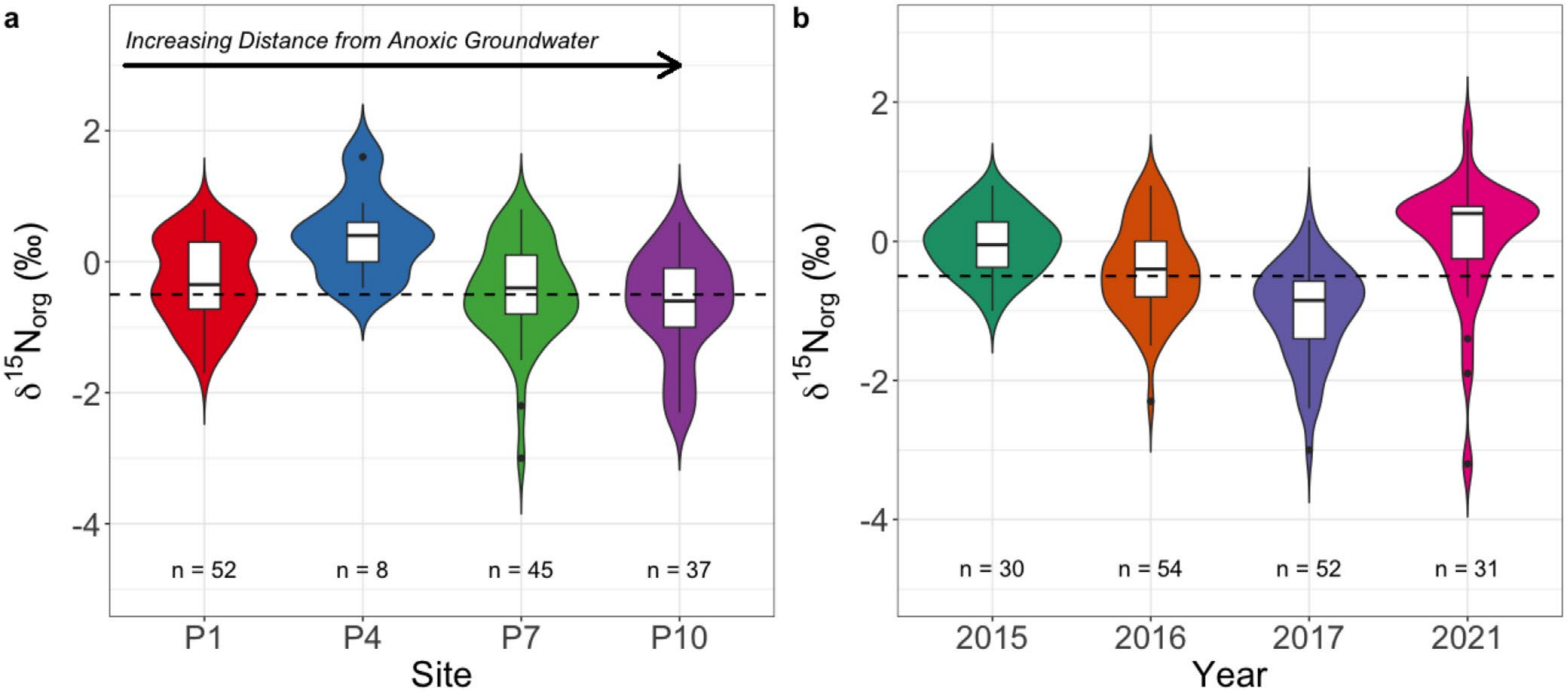
Distribution of MIS sediment δ^15^N_org_ values according to sampling (a) sites and (b) years. Intersite and interannual δ^15^N_org_ variability is statistically significant; however, individual years and sites tend toward an average (± standard deviation) δ^15^N_org_ value of −0.5‰ ± 0.8‰ (dashed line; *n* = 167). Sediments (*n* = 25) with no information regarding sampling collection sites were omitted from (a) but included in (b).

Anoxic sediments from MIS range from −3.2‰ to +1.6‰ for δ^15^N_org_ and from −28.8‰ to −20.9‰ for δ^13^C_org_. The sampling site and interannual variability at MIS is shown in Figure 4. Results of the pairwise Mann Whitney U‐test, in which each sampling site and year was compared against all others individually, reveal statistically significant differences in most cases, except for the P1 versus P7 site comparison (*p* = 0.30) and the 2015 versus 2021‐year comparison (*p* = 0.17) (Table S3). Despite intersite and interannual differences, average δ^15^N_org_ values of individual sites and years tend toward negative values (Table S4). With an average δ^15^N_org_ value of −0.5‰ ± 0.8‰, MIS sediments fall into the isotopically lighter range of both the entire modern lake dataset and the anoxic lake subset (average: +2.9‰ ± 3.2‰).

### 3.3 Ancient Lacustrine Sedimentary C and N Trends

**FIGURE 5:**
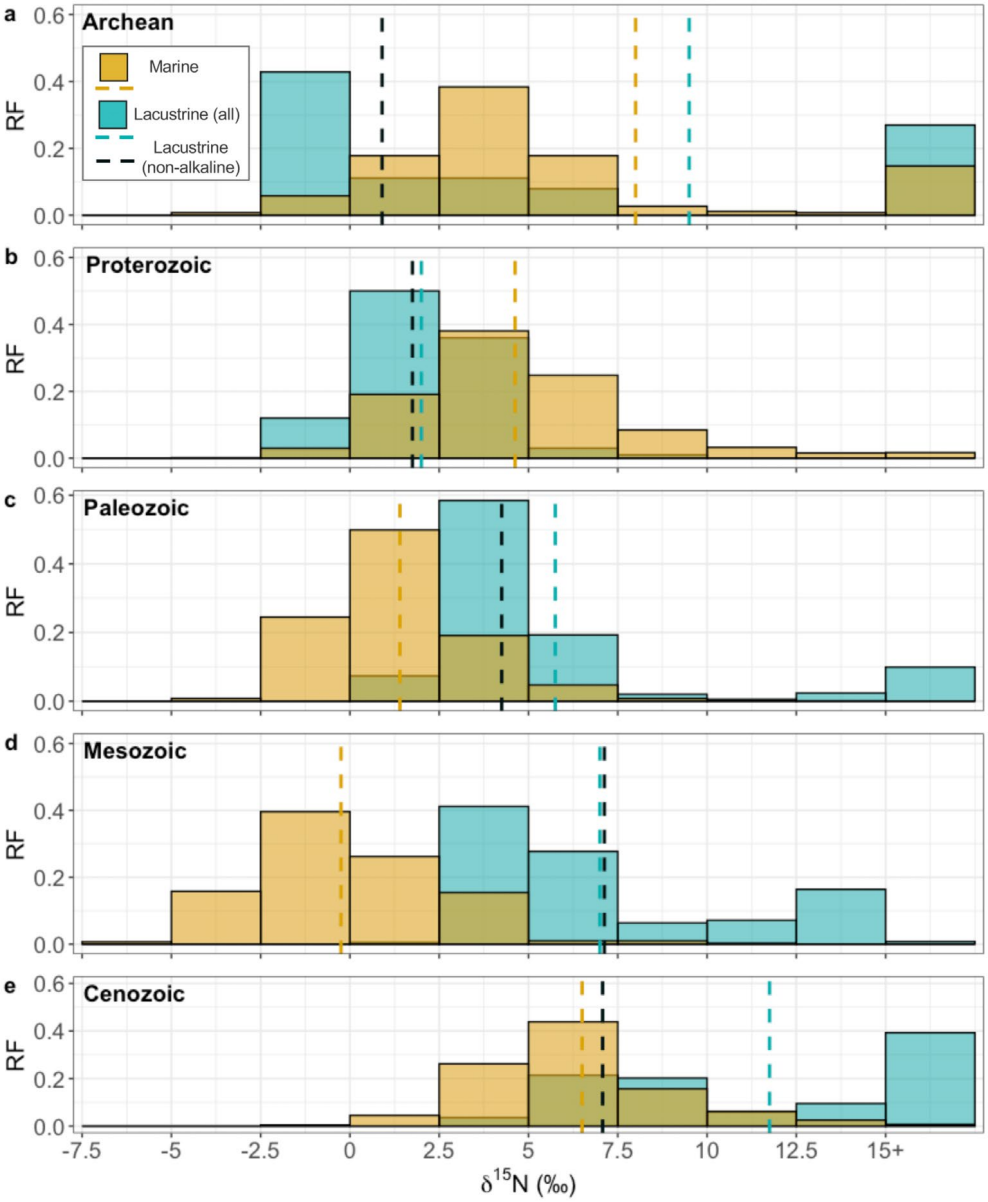
Relative frequencies (RF) of δ^15^N values from ancient marine sediments (gold) are shown alongside those from ancient lacustrine sediments (teal), with samples filtered to include only those of greenschist‐grade metamorphism or lower. Respective means are indicated by dashed lines, with the navy‐blue dashed line representing the average δ^15^N for non‐alkaline lacustrine sediments. Considering only non‐alkaline lacustrine units, the lacustrine δ^15^N distribution shows a distinct pattern compared to the marine, with a general shift toward higher δ^15^N values from the (a) Archean to the (e) Cenozoic. Enriched δ^15^N above 10‰ in lacustrine sediments are associated with alkaline conditions, according to the original publications (see Section 4.3 for discussion of high positive values).

**FIGURE 6:**
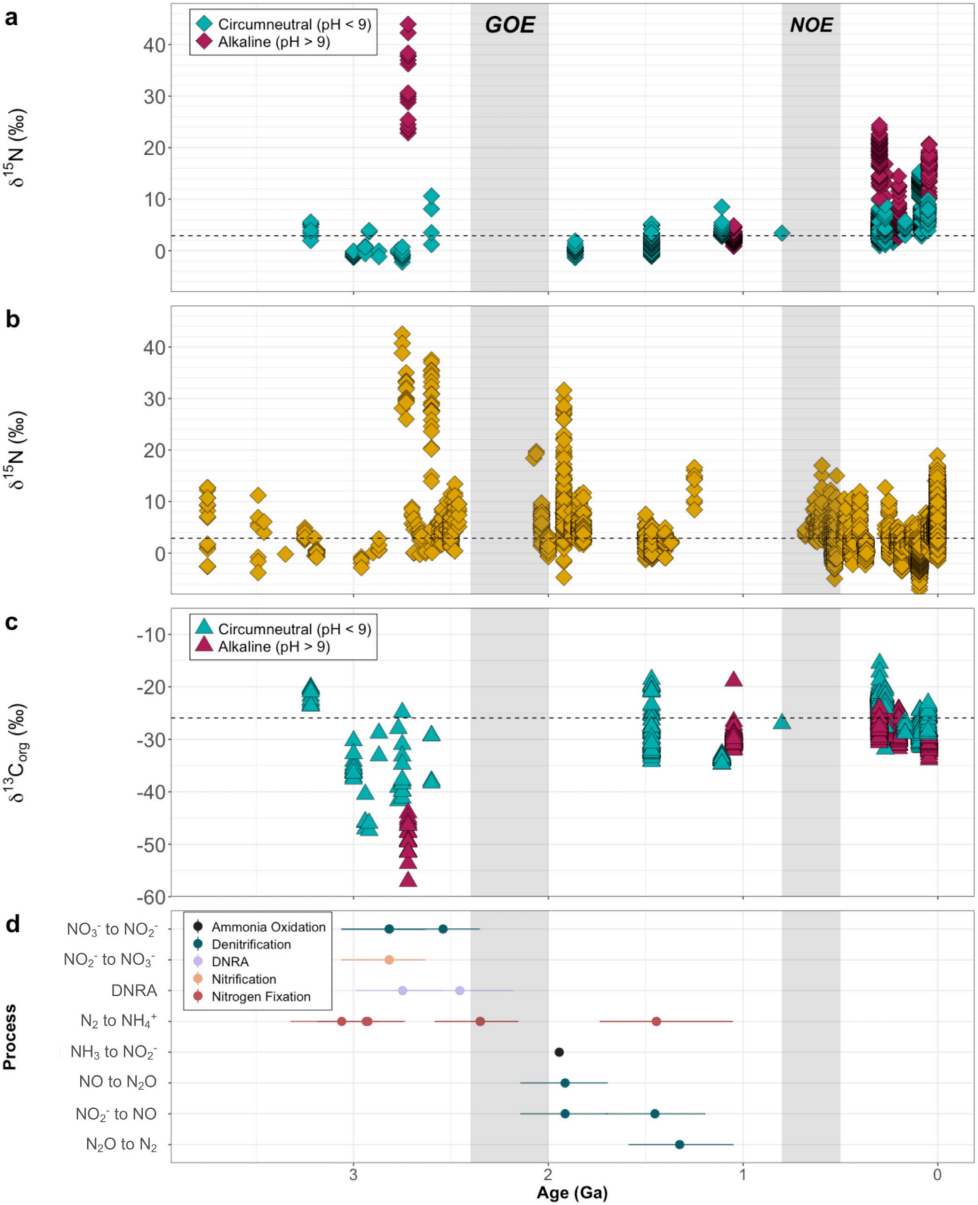
Geologic trends in nitrogen, organic carbon, and genes linked to nitrogen cycling with reference to the Great Oxygenation Event (GOE) and the Neoproterozoic Oxygenation Event (NOE) (vertical gray shading). Dashed vertical lines represent modern lake averages for δ^15^N: +2.9‰ and δ^13^C_org_: −25.99‰. (a) Compilation of published nitrogen isotope compositions of lacustrine sediments from the Archean to the Cenozoic (3.0 to 0.05 Ga), separated by alkalinity as specified in the original papers. (b) Marine sediment nitrogen isotope compositions from 3.75 Ga to present adapted from Stüeken et al. (2016), including all samples regardless of metamorphic grade. (c) Compilation of published organic carbon isotope compositions of lacustrine sediments from the Archean to the Cenozoic (3.0–0.05 Ga), separated by alkalinity as specified in the original papers. (d) Estimated earliest origin dates with 95% confidence interval for nitrogen cycling genes based on clock models (Liao et al. 2024; Parsons et al. 2021).

Ancient lacustrine sediments display an overall increase in δ^15^N from the Archean to the Cenozoic (Figure 5). Exceptions to the increasing δ^15^N trajectory throughout the geologic record include alkaline lakes (pH > 9) which consistently exceed mean δ^15^N values of circumneutral ancient lakes (alkaline: +11.4‰ ± 9.0‰; circumneutral: +5.0‰ ± 3.2‰) (Figure 6a). While the overall δ^13^C_org_ values of ancient and modern lakes show considerable overlap, ancient lakes include notably negative δ^13^C_org_ values as low as −57.06‰. Ancient lakes appear to be more positive for δ^15^N with an average of +6.3‰ ± 5.7‰ and maximum values as high as +44‰ (Table 1). Even non‐alkaline lakes from the relatively recent Cenozoic era show higher δ^15^N values with an average of +7.0‰ ± 1.4‰ compared to modern lakes (Figure 5). Ancient lakes also exhibit a positive correlation between %TOC and %TN as observed in modern lake sediments due to their association with organic matter (*r*_*s*_ = 0.70, *p* < 0.0001) (Table S5). However, a negative correlation between δ^15^N and δ^13^C_org_ is apparent in ancient lacustrine sediments (*r*_*s*_ = −0.13, *p* < 0.0001).

## 4 Discussion

### 4.1 Modern Lake Sediment C and N Isotopic Patterns

Lacustrine sedimentary geochemical records provide important insight into environmental conditions in comparison to marine settings. Modern lake sediment δ^15^N values (−21.9‰ to +17.0‰, mean: +2.9‰) are more negative than marine sediments (+2.5‰ to +16.6‰, mean: +6.7‰) (Tesdal et al. 2013), indicating differences in terrestrial and marine N sources and transformations. Unlike the oceans, which are deeper and have a higher buffering capacity, lakes and their sedimentary organic material are likely more strongly influenced by allochthonous inputs. For example, aquatic and terrestrial plant inputs to lacustrine sedimentary organic matter (median δ^15^N: +3.5‰; Chappuis et al. 2017) may result in lower δ^15^N values compared to oceanic values, where deep ocean nitrate (~ +5‰) is the primary N source for phytoplankton (Tesdal et al. 2013).

Within the modern lake dataset, water column oxygen conditions influence anomalous lake sediment δ^13^C_org_ and δ^15^N signatures. Although significant overlap exists between the isotopic signals of sediments from anoxic and oxic lake environments, hypolimnetic redox conditions result in some distinct isotopic trends. For example, low δ^15^N values (< −5‰) are only found in wholly oxic water columns, while low (< −32‰) or high (> −16‰) δ^13^C_org_ values are only found in wholly or partially anoxic water columns (Figure 3a). In anoxic lakes, anaerobic metabolic processes such as methanogenesis and methanotrophy can be of greater influence on sedimentary δ^13^C_org_ than primary production, resulting in as much as 30‰ discrimination and resulting in depletion of ^13^C in biomass (Garcia et al. 2021). Methanogenesis in anoxic lakes can be intensified by anthropogenic activity such as in the case of Lake Fischkaltersee, Germany and Lake Planina, Slovenia. Eutrophication in these lakes promotes and sustains anoxic conditions, leading to increased organic matter availability for methane production (Ogrinc et al. 2008; Braig et al. 2013). In our dataset, the anthropogenic influence on methanogenesis is marked by δ^13^C_org_ values below −30‰ from these two lakes (Figure 3b).

The very negative δ^15^N values observed in oxic lakes are more challenging to explain. The low δ^15^N values cannot result solely from N input via nitrogen fixation because this N‐transformation has minimal fractionation, typically producing organic matter with δ^15^N values near atmospheric N (−2‰ to +2‰; Quan and Falkowski 2009). Instead, the negative δ^15^N values may originate from a combination of anthropogenic pollution, natural processes like nitrification, or even the effects of invasive species.

First, pollution could cause negative δ^15^N values. The most negative δ^15^N values (< −10‰) in our dataset represent samples from the convergent gyre in southern Lake Michigan (Kerfoot et al. 2008), where materials from human industrial activities could accumulate and affect ^15^N ratios (Reavie et al. 2021). While synthetic fertilizers range between −2‰ and +2‰ (Bateman and Kelly 2007), industrial inorganic nitrogen discharge or microbial wastewater treatment can drive dissolved inorganic nitrogen δ^15^N values as low as −13‰ (Popescu et al. 2015).

Low δ^15^N values of NO_3_^−^ (and therefore by extension δ^15^N values of organic matter) are also typical in undisturbed ecosystems where nitrification is the dominant source of NO_3_^−^ (e.g., Mulholland et al. 2000). Complete nitrification only occurs in oxygenated water columns and sediments, and conventionally occurs in two steps: ammonium is first converted to nitrite (ammonia oxidation), followed by the oxidation of nitrite into nitrate (Prosser 1990; Ward and Jensen 2014). The combined isotope effects of these two reactions are only expressed if the reaction from ammonium to nitrite does not go to completion, resulting in isotopically depleted NO_3_^−^ (Casciotti et al. 2003; Casciotti 2009; Buchwald and Casciotti 2010). An example of a lake where nitrification likely contributed to low δ^15^N values is Lake Superior, which has average water column δ^15^N‐NO_3_^−^ values of −2.3‰ that result from high rates of nitrification coupled with lower rates of carbon burial and low rates of denitrification in oxygenated sediments (Finlay et al. 2007). Like Lake Superior, Lake Michigan's water column and sediments are frequently oxygenated, and therefore nitrification could be part of the explanation for the occurrence of low δ^15^N values.

Lastly, much of Lake Michigan has been heavily colonized by invasive dreissenid mussels. They have been shown to increase the sediment abundance of nitrifying taxa such as *Nitrospira* and *Nitrosomonas* that likely oxidize mussel‐excreted ammonium (Lee et al. 2015), which would result in a low δ^15^N signature.

### 4.2 Low N Isotopic Variability in Middle Island Sinkhole, Lake Huron

The low‐oxygen, iron‐rich, and sulfate‐rich waters of Middle Island Sinkhole, along with the widespread presence of microbial mats, make it a valuable analogue for Proterozoic ocean conditions (Lyons et al. 2009; Poulton and Canfield 2011; Rico et al. 2019). An estimate of how much isotope values could be expected to vary within a single site is important to constrain ancient sediment isotope records. We therefore evaluated intrasite variability between sampling locations and year of collection at MIS (Figure 4). MIS sediment δ^15^N_org_ values display a narrow range from −3.2‰ to +1.6‰ and overlap with reported phytoplankton δ^15^N values in Lake Huron, supporting prior conclusions that primary producers are the main source of organic matter to sediments (Nold et al. 2013). Seasonal and annual changes in local ice cover, chlorophyll levels, and precipitation significantly influence both the concentration and isotope composition of organic carbon in sinkhole sediments. Specifically, organic carbon concentrations are positively correlated with average chlorophyll, annual precipitation, and the mean annual lake surface temperature (Howard et al. in prep). Although groundwater strongly influences water column oxygen levels at MIS, its influence on sediment geochemistry seems minimal as reflected by minimal change in conservative ion concentrations with sediment depth (Kinsman‐Costello et al. 2017). Despite some spatial and temporal intrasite variability, sinkhole sediment isotopic signatures are overall reflective of organic matter source, which is primarily water column phytoplankton.

δ^15^N_org_ values for the entire MIS sediment collection span a range of 4.8‰, indicating low overall variability should be expected within a single lacustrine unit. Most ancient lacustrine units have δ^15^N less than +5‰, with a few exceptions exceeding +10‰. The Green River, Fencheng, Towaco, and Tumbiana formations—all alkaline units—exhibit high δ^15^N variability, with individual formation ranges (maximum minus minimum δ^15^N values) spanning from +10.6‰ to +21.2‰. This pronounced variability is likely due to severe ammonia degassing promoted by anoxic atmospheric and bottom water conditions, leading to amplified degassing and extremely high δ^15^N values relative to modern lakes experiencing this process (see Section 4.3 for further discussion).

### 4.3 High N Isotopic Values Record Alkalinity

While a marine origin for the Tumbiana Formation has been previously proposed (Thorne and Trendall 2001; Sakurai et al. 2005), several lines of evidence support deposition in an alkaline lacustrine setting, including the absence of sedimentary features reminiscent of tidal activity, mudcracks, and stromatolites reflective of evaporative/shallow waters (Stüeken et al. 2015), and rare earth element patterns comparable to other ancient alkaline lakes (Bolhar and Vankranendonk 2007; Coffey et al. 2013). Two explanations have been posited for the high δ^15^N values recorded by the Tumbiana Formation: (1) onset of nitrification coupled to removal of nitrite and nitrate (Thomazo et al. 2011) or (2) dissociation of NH_4_^+^ to H^+^ and volatile NH_3_ (Stüeken et al. 2015). However, molecular clock studies suggest that while some enzymes in the modern nitrification pathway predate the GOE (Figure 6d), the enzymes found in most modern clades of ammonia oxidizers, including bacteria (Liao et al. 2024) or archaea (Yang et al. 2021), likely emerged after the GOE. Although it is possible that the enzymatic machinery for bacterial ammonia oxidation originated earlier, this process may have been negligible, leaving minimal to no isotopic fingerprint in the sedimentary record (Ossa Ossa et al. 2022). Therefore, fractionation from loss of volatile NH_3_ may be the dominant process in these ancient alkaline (pH > +9) lakes. At a pKa of 9.25, dissolved NH_4_^+^ is converted to NH_3_ and H^+^, resulting in the degassing of NH₃ and isotopic enrichment of the remaining NH_4_^+^ due to Rayleigh distillation isotopic fractionation associated with ammonia volatilization. Although ammonia degassing is not restricted to anoxic environments, prior to the widespread oxygenation of Earth's atmosphere and oceans, anoxic conditions likely promoted this process by preventing ammonia removal through oxidation and facilitating ammonia accumulation and rapid release (Stüeken et al. 2015). Ammonia degassing is ultimately reflected by high sedimentary δ^15^N values (Li et al. 2012; Stüeken et al. 2015; Deng et al. 2018). While strongly positive δ^15^N values (+10‰ to +50‰) are also documented in the marine sedimentary record during the NIE, they are attributed to ammonia oxidation occurring under low, yet sufficient, oxygen concentrations (Pellerin et al. 2024). In particular, ammonia degassing would be expected to be more pronounced in enclosed systems such as lakes than in open shallow marine shelves. Strongly positive δ^15^N and highly negative δ^13^C_org_ values (< −40‰) as observed in the Tumbiana Formation may reflect methanotrophic ammonium oxidation under methane‐rich, hydrothermally influenced conditions, as proposed for the Neoarchean marine Manjeri and Cheshire formations (Martin et al. 2025). If such a process also occurred in the Tumbiana Formation, it could have amplified the positive enrichment imparted by ammonia degassing.

Under oxygenated atmospheric conditions, the dissociation of NH_4_^+^ to H^+^ and volatile NH_3_ was likely limited, which may explain the lower δ^15^N values observed following the Archean. The onset of the complete nitrification pathway post‐GOE may also offer an explanation for the relatively lower ^15^N values seen in later alkaline lakes relative to the Tumbiana Formation, as the combined fractionation of ammonia oxidation and nitrite oxidation could act to lower the ^15^N of the N substrates available to phytoplankton due to the inverse isotope fractionation of nitrite oxidation (Casciotti 2009).

**FIGURE 7:**
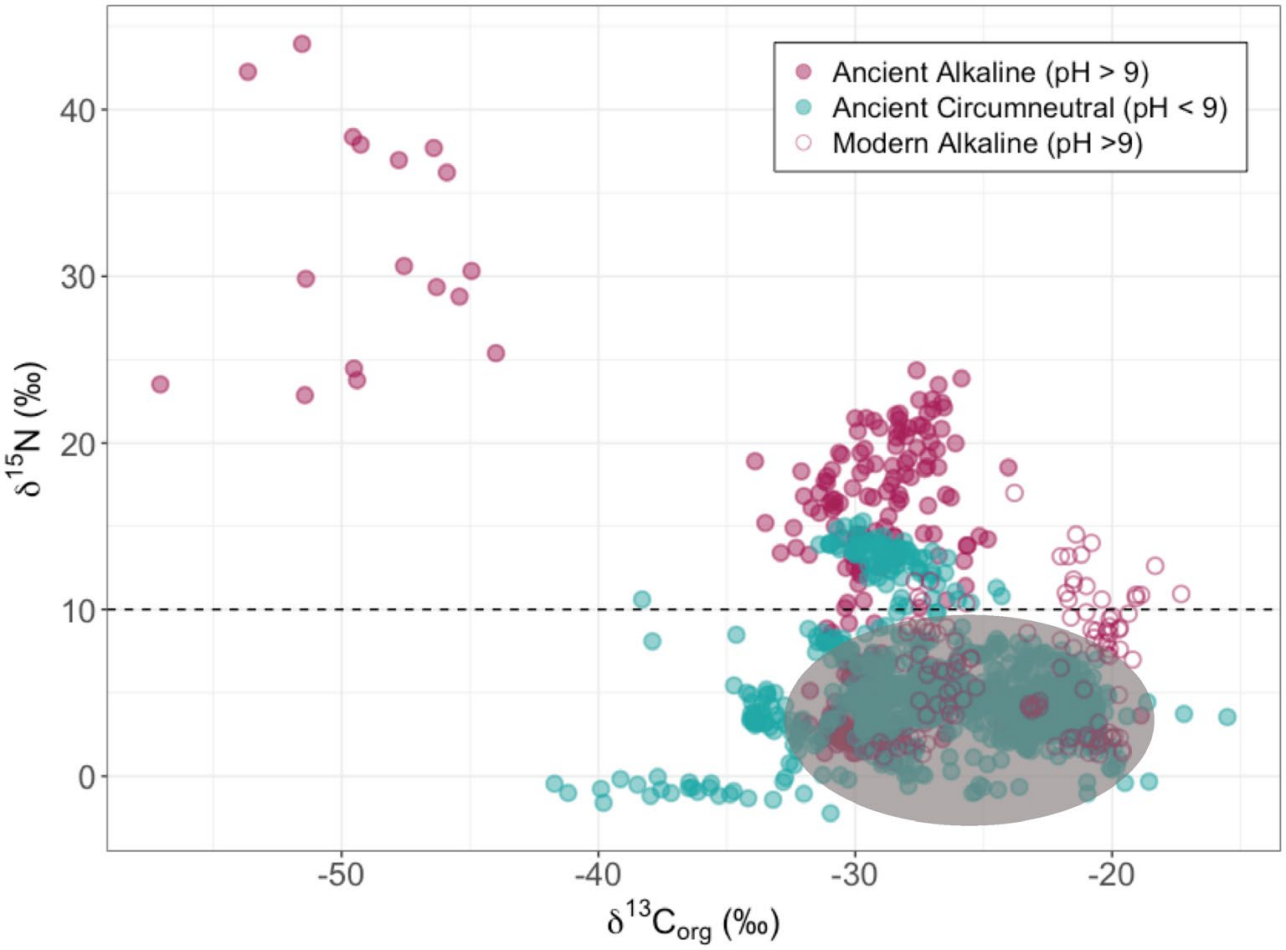
Sediment δ^15^N versus δ^13^C_org_ values for both ancient circumneutral (total samples = 986, geologic units = 27) and alkaline lakes (pH > 9) (total *n* = 264, geologic units = 6) in addition to the subset of modern alkaline lakes (total *n* = 109, sites = 7). Sediment δ^15^N values above 10‰ encompass most ancient alkaline lakes. The grey field represents the modern circumneutral 95% confidence array from Figure 3. Although both modern and ancient alkaline conditions drive δ^15^N enrichments, ancient lacustrine sediments show methanogenic‐like depleted δ^13^C_org_ values outside of the shaded grey modern data array.

In our dataset, all ancient and modern alkaline lakes, with the exception of Lake Tanganyika, exhibit some sedimentary δ^15^N values exceeding 10‰ (Figure 7), supporting the use of lacustrine sediment δ^15^N signatures as a basicity proxy, where values > +10‰ indicate alkaline lake conditions (Deng et al. 2018; Stüeken, Tino, et al. 2020; Xia et al. 2022). Other indirect proxies for determining the alkaline nature of an ancient lake include but are not limited to the presence of alkali minerals, nodular and bedded cherts, sedimentary evaporative features such as desiccation cracks, and the presence of stromatolites (Cao et al. 2020; Xia et al. 2021, 2024). For example, given that the maximum δ^15^N values of the Mesoproterozoic Arctic Bay Formation only reach +4.8‰, this unit may not have been alkaline or have maintained consistent alkaline conditions as posited by Hodgskiss et al. (2020). Furthermore, although a lacustrine origin for the Arctic Bay Formation has been suggested by osmium isotope ratios, there is also evidence of a degree of sustained marine input into the basin (Hodgskiss et al. 2020), which could have driven pH closer to marine values and thus minimized the extent of ammonia volatilization in the basin.

Climatic conditions, terrestrial inputs, and the presence of other nitrogen pathways should also be considered when interpreting δ^15^N signatures within single lacustrine units to determine the plausibility of alkaline conditions. For example, N cycling in Lake Bosumtwi, an African alkaline lake, is climate dependent, with warm periods inducing excess evaporation and pH increases leading to δ^15^N values > +10‰ while cooler periods are characterized by δ^15^N values < +10‰ (Talbot and Johannessen 1992). With careful assessment of thermal maturity, lipid biomarker studies of ancient alkaline lakes can be conducted to assess paleoclimatic variations (Luo et al. 2019; Xia et al. 2021). The dominance of other nitrogen cycling pathways can also lead to δ^15^N values < +10‰ in alkaline lakes. In Lake Tanganyika, anaerobic nitrogen fixation by cyanobacteria dominates in the surface waters under stratified conditions and introduces a very depleted δ^15^N source, leading to relatively low δ^15^N signatures preserved in sediments (Ehrenfels et al. 2023).

### 4.4 Geologic Record of C and N Isotopes in Lacustrine Sediments

Unlike the marine δ^15^N record, lacustrine sediments reveal a distinct overall shift toward enriched δ^15^N from the Archean to the Cenozoic, reflective of the rise in metabolic N‐diversity as a result of progressively increasing atmospheric oxygenation. Anoxic conditions prevailed during the Archean, as reflected by lacustrine δ^13^C_org_ values below −30‰, which coincide with modern strictly anaerobic methanotrophic signatures (Figure 6c). While δ^13^C_org_ alone is not a definitive proxy for oxygenation, it may reflect values associated with anaerobic metabolisms indicative of low‐oxygen conditions. Prior to the GOE, which marked the first widespread increase in oxygen in Earth's atmosphere ca. 2.45 Ga, marine (Mesoarchean [*n* = 46]: +1.1‰ ± 1.9‰; Stüeken et al. 2016) and circumneutral lacustrine (Archean [*n* = 44]: +0.8‰ ± 2.3‰) δ^15^N values fall within the range of Mo‐based nitrogen fixation (−2‰ to +1‰; Zhang et al. 2014), suggesting the early presence of a biologically mediated N cycle (Stüeken et al. 2016). Molecular clock dating estimates indicate that the genes essential to nitrogen fixation (e.g., nifH, nifD) appeared by the Mesoarchean, further supporting early microbial influence (Figure 6d; Parsons et al. 2021). Microbially influenced N cycling is further corroborated by the significant overlap of ancient lacustrine δ^15^N values to modern lake sediment δ^15^N values, with the exception of a singular alkaline lake, the Tumbiana Formation (Figure 6a, see Section 4.3) discussed above. The Neoarchean is marked by an increase in lacustrine and marine δ^15^N values that coincides with the presence of new nitrogen metabolic pathways (Figure 6; Parsons et al. 2021) such as nitrification. Nitrification, a process requiring oxygen, is among the metabolisms that appear later in the Archean and could have been linked to localized oxygen oases prior to the GOE (Planavsky et al. 2014; Kurzweil et al. 2016; Ossa Ossa et al. 2018, 2019). Modern nitrifiers can be found in marine oxygen minimum zones (Lam and Kuypers 2011) as well as at oxic‐anoxic transition zones in sediments (Mortimer et al. 2004), likely reminiscent of their appearance during a transition in oxygen conditions.

Although lacustrine and marine δ^15^N records are scarce during the interval of the GOE, we can use Proterozoic and Phanerozoic records to infer changes to the N cycle linked to global oxygenation. Several Proterozoic basins in our dataset have been proposed as having a marine influence, and interpretations of their depositional environments remain debated. In the Nonesuch Formation, sedimentary features interpreted as tidal currents (Jones et al. 2020) and trace element signatures (Stüeken, Jones, et al. 2020) were used to suggest mixing between lake and marine waters in an estuarine environment. However, a recent reconstruction of the paleoenvironment of the Nonesuch Formation, with a broader suite of evidence including mineralogy, stratigraphy, tectonic context, paleogeography, and palynology, strongly supports deposition in a shallow lacustrine environment with oxygenated surface waters and reduced oxygen at depth (Slotznick et al. 2023). Additionally, given current plate reconstructions for the Mesoproterozoic (Li et al. 2023), the Nonesuch Formation was deposited > 1000 km from the open ocean, making marine incursions into the basin unlikely. So, we retain that the Nonesuch Formation is lacustrine but recognize that its deposition is potentially ambiguous. In the Belt Supergroup, while the sedimentology has been interpreted as supporting a lacustrine environment (Winston 1991), microfossil assemblages suggest a shallow marine environment (Adam et al. 2016). While the depositional environment of these basins remains debated, they potentially capture the dynamic connections between lakes and oceans in the Proterozoic, a time also marked by fundamental shifts in oxygen levels and microbial life.

Anoxic water column conditions below oxygenated surface waters prevailed in marine settings until the Neoproterozoic (Hood and Wallace 2014), but an increase in lacustrine δ^13^C_org_ values immediately following the GOE suggests the rise of oxygen in lacustrine environments coincident with another terrestrial record, the paleosol chromium isotope record, which suggests a shift to oxidative weathering (Colwyn et al. 2019). Thus, the shallower depth of water in lakes may have allowed for deeper oxygen penetration than in marine settings. Between the GOE and the Neoproterozoic Oxygenation Event (NOE), ca. 0.85 Ga, δ^13^C_org_ values range from −10‰ to −30‰, potentially reflecting the rise in diversity of cyanobacteria and eukaryotic algae alongside increases in oxygenation in the atmosphere and shallow bodies of water (Garcia et al. 2021). It is also during this time period that additional genes associated with denitrification and nitrogen fixation appear. Denitrification, though an anaerobic process, likely appeared after widespread nitrification supplied sufficient nitrate. Metabolic processes such as denitrification could therefore be responsible for increasing δ^15^N in lacustrine sediments through time.

## 5 Conclusions

Lacustrine sediment δ^15^N records offer additional insight into the progression of the N cycle throughout Earth's atmospheric and biological changes. The sensitivity of lakes to early atmospheric changes is evident in the enriched δ^15^N values of lacustrine sediments through time. Modern anoxic and oxic lake δ^15^N and δ^13^C_org_ thresholds could be useful for determining ancient depositional environments where water column oxygen conditions are uncertain. Alkaline (pH > 9) conditions can also be reliably deduced from high δ^15^N of > +10‰ found in both modern and ancient lake sediments. Lakes represent dynamic interfaces between terrestrial and aquatic systems; therefore, understanding how early atmospheric changes, such as oxygenation, impacted them can provide insight on the progression of both the N cycle and the evolution of life.

## Conflicts of Interest

The authors declare no conflicts of interest.

## Supporting information


**Data S1:** gbi70033‐sup‐0001‐DataS1.xlsx.

**Data S2:** gbi70033‐sup‐0002‐DataS2.xlsx.

**Data S3:** gbi70033‐sup‐0003‐DataS3.xlsx.

**Data S4:** gbi70033‐sup‐0004‐supinfo.docx.

## Data Availability

The data that supports the findings of this study is available in the S4 material of this article.
