# Supplementary material for: Oxygenation and Alkalinity Drive the Lacustrine Nitrogen Isotope Record Throughout the Past 3.2 Billion Years
Source: Geobiology. 2025 Sep 18;23(5):e70033. doi: 10.1111/gbi.70033 (PMC12444620; doi:10.1111/gbi.70033)
Supplement: Supplementary file 4 — Data S4: gbi70033‐sup‐0004‐supinfo.docx. [file GBI-23-e70033-s002.docx]

**Oxygenation and alkalinity drive the lacustrine nitrogen isotope record throughout the past 3.0 billion years: Supplemental Material**


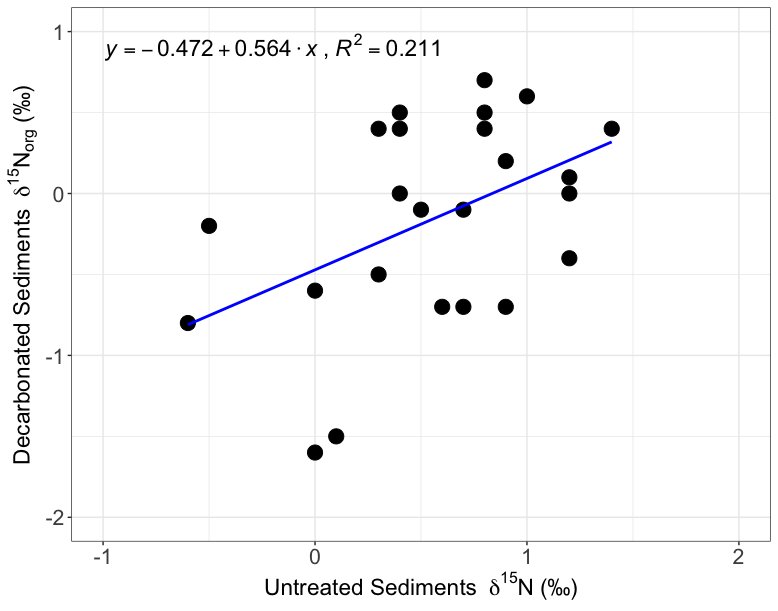


**Supplementary Figure 1:** Comparison of replicate δ^15^N measurements from decarbonated and untreated Middle Island Sinkhole sediments.

**Supplementary Table 1:** Minimum, maximum, and average (± standard deviation) total organic carbon (%) and total nitrogen (%) for all modern lakes with both geochemical measurements available (sites *n* = 163, total samples *n* = 1353). Sedimentary geochemical data for the full modern dataset is shown along with the subset of anoxic (sites *n* = 18) and oxic (sites *n* = 145) lakes.

|  |  | TOC (%) | TN (%) |
| --- | --- | --- | --- |
| Modern Lakes  (n = 1353) | Min. | 0.05 | 0.00 |
|  | Max. | 30 | 3.4 |
|  | Mean & S.D. | 6.09 ± 5.54 | 0.53 ± 0.45 |
| Anoxic Lakes  (n = 593) | Min. | 0.26 | 0.00 |
|  | Max. | 30.00 | 3.40 |
|  | Mean & S.D. | 7.57 ± 5.62 | 0.64 ± 0.48 |
| Oxic Lakes  (n = 760) | Min. | 0.05 | 0.00 |
|  | Max. | 28.27 | 2.52 |
|  | Mean & S.D. | 4.93 ± 5.19 | 0.44 ± 0.41 |

**Supplementary Table 2.** Spearman correlation coefficients between total nitrogen (%), total organic carbon (%), nitrogen isotopes (‰), and organic carbon isotopes (‰) of modern lake sediments (sites *n* = 163, total samples *n* = 1353). Values in bold are significant (p < 0.05), while bold and underlined values are highly significant (p < 0.0001).

| Variables | %TN | δ^15^N | %TOC | δ^13^C_org_ |
| --- | --- | --- | --- | --- |
| %TN | 1 |  |  |  |
| δ^15^N | **-0.33** | 1 |  |  |
| %TOC | **0.93** | **-0.32** | 1 |  |
| δ^13^C_org_ | 0.05 | **0.10** | **0.11** | 1 |

**Supplementary Table 3:** Results of the pairwise Mann-Whitney U-Test of Middle Island Sinkhole, Lake Huron δ^15^N sediment values from the sampling sites and sampling years. Results that reach empirical level of significance: p < 0.05 are in bold type.

|  |  | *p*-value |
| --- | --- | --- |
| Sampling Site Pairs | P1 vs. P4 | **0.016** |
|  | P1 vs. P7 | 0.2966 |
|  | P1 vs. P10 | **0.04726** |
|  | P4 vs. P7 | **0.005878** |
|  | P4 vs. P10 | **0.001624** |
|  | P7 vs. P10 | **0.005878** |
| Sampling Year Pairs | 2015 vs. 2016 | **0.006709** |
|  | 2015 vs. 2017 | **1.239 x 10^-9^** |
|  | 2015 vs. 2021 | 0.1692 |
|  | 2016 vs. 2017 | **3.474 x 10^-5^** |
|  | 2016 vs. 2021 | **0.001726** |
|  | 2017 vs. 2021 | **3.77 x 10^-8^** |

**Supplementary Table 4:** Middle Island Sinkhole, Lake Huron δ^15^N average (± standard deviation) for each sampling site and sampling year.

|  |  | δ^15^N (‰) |
| --- | --- | --- |
| Sampling Site | P1 | -0.3 ± 0.6 |
|  | P4 | 0.4 ± 0.6 |
|  | P7 | -0.5 ± 0.8 |
|  | P10 | -0.6 ± 0.8 |
| Sampling Year | 2015 | 0.0 ± 0.4 |
|  | 2016 | -0.4 ± 0.7 |
|  | 2017 | -1.0 ± 0.7 |
|  | 2021 | 0.0 ± 0.9 |

**Supplementary Table 5.** Spearman correlation coefficients between total nitrogen (%), total organic carbon (%), nitrogen isotopes (‰), and organic carbon isotopes (‰) of ancient lake sediments (units *n* = 24, total samples *n* = 1002). Values in bold are significant (p < 0.05), while bold and underlined values are highly significant (p < 0.0001).

| Variables | %TOC | δ^13^C_org_ | %TN | δ^15^N |
| --- | --- | --- | --- | --- |
| %TOC | 1 |  |  |  |
| δ^13^C_org_ | 0.01 | 1 |  |  |
| %TN | **0.70** | **0.00** | 1 |  |
| δ^15^N | **0.07** | **-0.13** | **-0.07** | 1 |
